# Fever

**Published:** 1830-04-01

**Authors:** 


### FEVER.

1. A Treatise on Fever. By *Southwood Smith*, M.D. Physician to the London Fever Hospital. Octavo, pp. 436. 1830.

2. Clinical Illustrations of Fever, comprising a Report of the Cases treated at the London Fever Hospital, 1828-9. By *Alexander Tweedie*, M.D. Physician to the Fever Hospital, &c. Octavo, pp. 204. 1830.

We class these two publications together, not to compare nor yet to contrast them, for they will not bear such—but because they treat of the same subject—emanate from the same source—and are written by colleagues of the same institution. Yet they are very different works. The first is a regular systematic Treatise on Fever—the second little more than an extended Hospital Report; such as might and should be occasionally sent forth from an institution like the Fever Hospital—and is, we believe, the harbinger of some such publication as the first on our list. Considering the number of works on fever which we have grappled with and reviewed in this Journal during the last twelve years, it will not be wondered at that the sight of two new heads of the Hydra, almost at the same instant springing forth, should have occasioned something like the feeling of depression which a weary traveller experiences, on beholding from the summit of a mountain, which he thought was the last of the day's journey, a long and dreary ascent up an interminable chain of other and still higher ridges! We regret, and yet it is a selfish regret, that our readers cannot completely sympathize with us on such occasions as these. We have been more than once mortified, on taking up the Medico-Chirurgical Review, many hundred miles from the metropolis, and observing the pages of *fever articles* uncut, while all the rest of the sheets were sullied by careful thumbing and perusing—articles, too, which have cost us many hours of intense intellectual labour, while our brethren were sleeping soundly in their beds, or labouring in bringing forth productions of a much more lively kind. But, in our voyage through life, we must take the rough with the smooth—the adverse with the propitious gale—the frowns with the smiles of fortune. It would be ingratitude, as well as affectation, to say that we have peculiar cause to complain of the buffetings and storms of fate. On the contrary, we believe that our *wishes* to please and benefit our readers have often received that cheering reception which more splendid *powers* are entitled to expect.

The cloud of mystery which has hung over the subject of fever for three thousand years—the clashing, discordant, and endless theories which have been broached respecting its nature—the diametrically opposite modes of practice which have been advocated by the most renowned physicians of the ages in which they lived—the difficulties, not to say the dangers, of applying doctrines which have been engendered in the closet to diseases occurring in the chambers of the sick—these and many other circumstances may account for the apathy with which books on fever are received by the public, and explain the reason why the reviews of such books have been passed over unread, while light and miscellaneous articles have been carefully perused. A knowledge of these things might well tempt a journalist, who studies the taste of the age rather than the duties of his avocation and the solid interests of medical science, to slur over such productions, however meritorious in themselves, in order to give ampler space to subjects more excitant to the passions, or more gratifying to the propensities of human nature. But this practice we shall not follow.

The history of fever and of fever writers forbids all expectation of any important and sudden discovery, either in the theory or treatment of that disease, by the most gifted individual. The greater number of writers, indeed, and especially those master spirits who left doctrines as well as facts on record, have rather added to the mass of error than assisted in eliciting truth, in relation to fever. Hence, the modern inquirer has the difficult task before him of clearing away a vast heap of rubbish before he can lay even the foundation of a solid edifice. The excellent opportunities of studying the phenomena of fever at the Fever Hospital, and the known zeal and talents of the two authors who have now come forward, induce a rational hope that their observations will at least tend to render the line of demarcation between truth and error more distinct than it has hitherto been. The fixing of such a boundary would be a great benefit conferred on the profession, and we really believe that the works on our table will contribute to that desired event. We shall now endeavour to convey to our readers a concise view of what they are to expect from a more extended perusal of the originals.

Dr. Smith's work is divided into nine chapters, all of which we shall not equally examine, as they are rather unequal in interest.

The first chapter, as usual, is employed in the work of demolition rather than of construction. The various doctrines relative to the nature and seat of fever, from Hippocrates down to Broussais, are examined—and, we need hardly say, disposed of.

"The prevailing doctrines relative to the nature and seat of fever at present, then, are two, the direct reverse of each other; one, that it is a general disease affecting the entire system; that this affection of the system consists of debility which is manifested first in a loss of energy of the brain, but which rapidly extends to every organ and every function, and that, consequently, *the absence of any primary local disease*, ought still to form, as it has so long formed, an essential part of the definition: the other, that it is in the strictest sense a local disease; that its primary seat is invariably fixed in some one organ; that the affection itself consists of inflammation; and that that inflammation is seated, according to one opinion in the brain; according to the other in the stomach." 27.

The first doctrine above alluded to is, we think, not quite fairly stated. Those who look upon fever as a general disease, and who believe that it, at the beginning, manifests loss of energy in the brain, do not stop there, as Dr. Smith has made them stop. They know well, that when reaction is fairly established, some organ usually becomes topically affected, that topical affection being usually inflammation, or something very like inflammation. This makes an important difference. The error in the foregoing statement has led Dr. S. into another mistake, that of supposing that the class of theorists above-mentioned, condemn all depletion as detrimental to the disease.

"The advocates of the first deprecate all active interference: the grand evil to be contended with is debility: the physician can easily weaken, but he cannot easily strengthen; he can depress to any extent he desires, but he cannot communicate power as he wishes. In a malady therefore of which the very essence consists in loss of energy the main duty of the physician is to husband the strength of the patient with the most anxious care, this being the chief means, as Cullen expressively termed it, of obviating the tendency to death. *The important inference is, that every kind and every degree of depletion that can add to the primary cause of the malady, must be abstained from with the utmost caution.*" 28.

We appeal to our readers whether the passage marked in Italics is not an overstrained representation of the practical views taken by the class in question. It is true that one or two writers—Dr. Stoker for example, have supported such a view of fever; but such is not the general principle acted on by practitioners in this country. Neither do the advocates for topical inflammation, as the cause of fever, unsheath their lancets and deplete away, as if they had pure phrenitis or enteritis to deal with. Even Dr. Clutterbuck lays down some restrictions on depletion in the shape of "modifications arising out of the *peculiar* nature of the organ affected, and, in some degree also the *nature of the exciting* cause." We agree, however, with Dr. Smith, that the doctrines of Clutterbuck and Broussais must naturally lead the disciples of those teachers to treat fever and inflammation in the same manner.

So far this second sect is much more dangerous, in the application of its doctrines, than the other.

Dr. S. deplores, and well he may, "the degree in which the science of mind is neglected in our age and country—especially in our profession"—hence the miserable state of the "art of reasoning" among its members. Dr. S. conceives that all the partial and imperfect views of fever which have, from time to time, been brought forward, originate in one or other of the following errors:—

"Either that of assuming as a fact what is merely a conjecture; or that of assigning to the genus what belongs only to the species; or that of characterising the disease by what appertains only to a stage; or that of mistaking the effect for the cause. On careful examination it will appear that one or other of these errors, which are as serious as they are palpable, has vitiated in a greater or less degree every generalization of fever that has hitherto been attempted.

"Thus the believers in debility derive their notion of the whole disease from the phenomena which occur in the first and the last stages only: in these, it is true, they may find abundant evidence of debility: but then they overlook the intermediate stage in which there are generally the most unequivocal indications of increased sensibility in the nervous and increased action in the vascular systems: in this manner they characterise the disease by what appertains only to certain stages of it. Again, when they contend that debility is not only the essence of fever in general, but is really characteristic of every type of it, they affirm what is indisputable of fevers in particular seasons, in particular climates, or in particular constitutions; but beyond this their generalization cannot be extended: in this manner they assign to the genus what belongs only to the species. And when Cullen goes on to affirm that the proximate cause of all the morbid phenomena is a 'spasm of the extreme vessels,' he commits the additional and more palpable, but not less common error, of assigning as an undoubted fact, as a real and ascertained occurrence, what is only a conjecture, and for which there is not, and for which he does not even attempt to adduce the shadow of evidence.

"Precisely similar to this is the error of those who for the most part belong to the same school, and who attribute the essence of fever to a morbid condition of the blood. The blood may be diseased in fever, but if it be so, these writers do not *know* it, or at least they do not adduce any evidence that they are in possession of such knowledge: they do not appear so much as to have questioned chemistry; at all events, it is certain that they have hitherto received no satisfactory answer. There is no evidence on record that the alleged deterioration of the blood takes place in every type and every degree of fever: and if there were it would still be but one event among many, and one that occurs late in the series, and therefore could possibly be nothing more than an effect.

"In like manner those who maintain that inflammation of the brain is the sole cause of fever, assume as an established and admitted fact the universal and invariable existence of inflammation of the brain in this disease. Inflammation of the brain, without doubt, is demonstrable of many individual cases, and of some whole types: but beyond this there is no proof that the generalization can be carried: the evidence indeed in regard to many cases is entirely against the assumption, and is as complete as negative evidence can well be: consequently it must be admitted that even this hypothesis, in the present state of our knowledge, is founded on the error of assigning to the whole genus what belongs only to particular species: and it would be trifling with the reader to attempt to prove, that this is still more certainly and strikingly true with regard to inflammation of the mucous membrane of the stomach and intestines—an affection which in innumerable cases in which its existence is certain, clearly appears on the slightest examination of the succession of events, to be an effect and not a cause." 33.

These extracts will lead our readers to expect some sober and rational views of fever from Dr. Smith, and we think they will not be disappointed. The first thing to be done in the investigation of fever, is to ascertain the concourse of symptoms—and the second, to determine the order in which they occur. This done, our author thinks the essential will be easily distinguished from the adventitious—the causes from the effects. It is not quite easy, however, to say what are the essential symptoms in a disease presenting such a variety. "Our guide is *invariableness* of occurrence."

"If we can ascertain that a certain number of events *invariably* take place in every form and every degree of fever, these events will give us the particular phenomena which are common to all the varieties of the disease. If we can further ascertain that these events *invariably* concur in a certain order, we shall have discovered what events bear to each other the relation of cause and effect. And the establishment of this relation of events, this constant connexion with each other, this uniform antecedence and sequence appears to me to be the only theory after which it is consistent with the principles of sound philosophy to search." 37.

Dr. Smith has endeavoured to establish this "constant connexion," carefully restricting himself to the deduction of legitimate conclusions from facts previously ascertained.

Whatever be the agents or exciting causes of fever, he observes, they can only induce the disease by effecting certain changes in certain organs. What then are the organs implicated? What the conditions thus induced in them? Which are the organs that sustain the first assault, which are those attacked in succession? The pathology to be laid before the reader will, he thinks, demonstrate the first two points—the establishment of the last two will be attempted by an examination of the histories of the cases. "It is only by observing the symptoms during life, and comparing them with the morbid appearances after death, that we can discover the signs which indicate the existence of these states." No doubt this is our only way:—but then, unless we had the opportunity of seeing the post-mortem changes at every period, from the first symptom till the last breath, how can we be certain of their nature? We have not these opportunities, for evident reasons, because people do not die of fever till the disease has acquired a certain degree of intensity. Then we see only the changes corresponding with the late, not with the early symptoms.

In the second chapter our author pursues the subject still more rigidly. Fever, he observes, is a genus consisting of several species, each species presenting many varieties. The external characters of these varieties and the internal states on which they depend, are so opposite, that no two diseases in any two parts of the nosological catalogue, present a more diversified appearance, or require a more varied treatment, than may be the case in two different types of fever. The fever of one country or one season is not the same as the fever of another country, or another season of the same country. Yet amidst this astonishing diversity, there is a something which preserves the identity, under every aspect which it has hitherto been observed to assume. Thus the term fever has never been denied to the disease as it occurs in this country—in the West Indies—at Batavia—and in Grand Cairo. Yet if four people labouring under these respective forms could be brought together in the ward of an hospital, what a difference would be seen in their exterior characters! What is this something which is common to all the forms of the disease? Boerhaave collected all the symptoms recorded by authors or observed by himself in fevers, and then threw out, one by one, those which did not appear in all cases of the disease. He was surprised to find himself, in the end, reduced to the small catalogue of three—shivering, frequent pulse, heat. And yet there is not one of these three that is invariably found in every type of fever—while, in innumerable instances, the combination of the three is absent. Cullen's definition is still less tenable, and our author thinks that the last item in it, "without any primary local affection," has a direct tendency to mislead the mind, and prevent it from observing the real phenomena of the disease. The fact is, as we have often maintained in this Journal, that fever is not an entity, but a series of events, which must for ever defy the narrow boundary of a definition, within which nosologists endeavour to confine it. The first object of enquiry is, what are the events which invariably occur in fever?

"Where shall we look for the events? Not in the symptoms. Symptoms are not events; they are only indications of events: symptoms depend upon states of organs; they are the external and visible signs of internal, and, for the most part, as long as life continues, invisible conditions. It is then to the state of the organs that we must look for the events of which we are in search.

"Are there any states of any organs that always exist in fever? Are the states constant? Are the organs affected constant; and can both be ascertained? If this can be truly answered in the affirmative; if it can be proved that there are certain conditions of certain organs which invariably exist in fever, in every type, in every degree, in every stage of it, we shall have arrived at a satisfactory conclusion relative to the first part of our inquiry.

"The evidence is as complete as observation during life and inspection after death can make it, that a morbid change does take place in a certain number of organs in every case of fever, from the most trivial intermittent to the most alarming continued fever, from the mildest plague to the most malignant typhus; that at the two extremes of this scale, and at all the intermediate gradations of it, there are certain organs which are always affected, and that the affection in all is similar.

"The identity of the organs is inferred from the indications they give of disordered function during life; the identity of the affection is inferred from the similarity of morbid appearances which they exhibit on examination after death.

"The organs affected are those which constitute the nervous system; those which constitute the circulating system, and those which constitute the systems of secretion and excretion. The spinal cord and the brain; the heart and the arteries, especially their capillary extremities; the secreting and the excreting organs, which in fact are composed, essentially, of the capillary extremities of the arteries; the secreting and the excreting extremities of these arteries, especially as they terminate in the external skin, and in the mucous membranes, which form the internal skin, this is the chain of diseased organs: derangement in the nervous and sensorial functions: derangement in the circulating function: derangement in the secretory and excretory functions, this is the circle of morbid actions.

"There never was a case of fever in which all these organs and affections were not more or less in a morbid state: there never was a concurrence of this morbid state, in this complete circle of organs, without fever. *The events which* invariably *concur in fever, then, are a certain deviation from the healthy state in the nervous and the sensorial functions; a certain deviation from the healthy state in the circulating function; a certain deviation from the healthy state in the functions of secretion and excretion.* A deviation from the healthy state in one circle of actions will not present the phenomena of fever; a deviation from the healthy state in two circles of action will not present the phenomena of fever; there must be a deviation in the three circles before fever can exist. Such then are the common phenomena of fever.

"For obvious reasons the detail of the proof that these several events really and invariably take place, must be postponed until the phenomena themselves have been stated, or what is termed the history of the disease has been given.

"But it is not the invariable concurrence of a particular number of events that is alone sufficient to constitute fever; to this must be added invariableness of concurrence in a particular order. As will be shewn in the proper place, there is complete and irresistible evidence that these events do occur in one invariable order. Derangement in the functions of secretion and excretion never comes first in the series; derangement in the nervous and sensorial functions never comes last in the series; derangement in the function of the circulation never comes either the first or the last in the series, but is always the second in succession.

"The order of events then is first, derangement in the nervous and sensorial functions; this is the invariable antecedent: secondly, derangement in the circulating function; this is the invariable sequent: and thirdly, derangement in the secreting and excreting functions; this is the last result in the succession of morbid changes." 50.

We appeal to our readers—we appeal to Dr. Smith himself, whether the above theory be not a mere version, rather more diluted with words, of the theory propounded in the following passage of a work published many years ago by the Editor of this Journal.

"*Ratio Symptomatum.*—We now proceed to trace the *action* of these febrific causes on the human frame—or in other words, the *ratio symptomatum* of fever itself; for in nature and in truth, there is no such thing as a *proximate cause* of this disease, the whole train of symptoms being a series of causes and effects, extremely difficult to delineate or comprehend. If any thing could deserve the name of *proximate cause*, it would be some peculiar state or phenomenon *invariably present* at the beginning of fever, and without which, the disease could not be said to exist. But all writers agree that there is no *one* symptom, state, or phenomenon which is constantly observable in fever. Neither quickness of pulse—increased heat—thirst—nor headache can be laid down as pathognomonic; for although *some* of these are *always* present, no *one* of them is invariably so.

"If an appeal, however, be made to accurate clinical observation, it will probably be found that from the first till the last moment of fever, *two phenomena* are constantly present—a derangement in the balance of the *circulation*, and of the *excitability*. If the calibre of the radial artery, or the strength and velocity of its pulsations shew nothing preternatural, (which by the bye will be a rare occurrence) yet, the experienced physician can instantly detect the unequal distribution of the vital fluid, as well by the torpid state of the *extreme* vessels on the surface, and throughout the glandular system, as by the turgidity of the *primary* trunks. The imperfect perspiration and secretions will point out the one; the peculiar febrile anxiety—hurried respiration on attempting to sit up or move—fulness of the præcordia, and heaviness about the head, will clearly demonstrate the other. In no one instance, during a long acquaintance with fever, have I failed to notice these indications of a deranged balance of the *circulation*.

"The proofs of broken balance in the *excitability* are equally manifest. It is now well known how much the functions of the glandular system are dependent on the nervous. In fever, the secretions are never perfectly natural. They are in general scanty—sometimes preternaturally copious; but always depraved. While this torpor or irregularity is going on in the glandular system, the nerves of sense shew plain marks of inequilibrium of excitability. The same degrees of light and sound that in health would be pleasing, will, in fever, be either distracting, or incapable of making any impression at all. The stomach will be in a state of morbid irritability, and the intestinal canal completely torpid. Speaking generally, however, the glandular or secreting system is irregularly torpid—the nervous or sentient system, irregularly irritable and debilitated.

"Now if we find that the general operation of the various *predisposing* causes of fever, is to disturb more or less, according to the force and condition of the subject, the balance of the circulation and excitability, we advance one step nearer to a knowledge of this *proximate cause* in fever, because we find in it the same *ratio symptomatum* as in all the phlegmasiæ, modified only by the *exciting* cause."*

In the year 1819, Dr. Wight published a paper on the nature of fever, in this Journal, in which he comes to the same conclusions as Dr. Smith. The following short extract will suffice to shew the similarity, if not identity of opinions.

"When Cullen says, 'that there is lesion of several functions,' he approaches the truth, but still the idea conveyed is imperfect; for if any part of the body is exempt, it is plain that there is present, not a general affection or disease of the whole body, but that of a part, or a local one; and this cannot be called fever, which spares none of the functions. If, then, we see that all the functions, vital, animal, and natural, are disordered in all cases, however much they may differ from one another, in manner, duration, appearance, and danger;

* Johnson on Tropical Climates, 2d Edit. p. 24.

if, in fine, such disturbance of the functions is present in every fever, have we not obtained a certain universal character by which fever is to be distinguished from every other disease?"* 312.

If these passages diminish the intensity of originality in Dr. Smith's doctrine, they will not probably detract from its stability or truth. Dr. Smith has also the merit of giving a greater development and illustration to the doctrine than any preceding writer. The following passage will shew that our author is not a disciple of either Clutterbuck or Broussais.

"No other disease exhibits the same train of phenomena in the same order of succession. In inflammation some of the phenomena are the same: but the order in which they concur is not the same; and this affords a clear and universally applicable mark of distinction between fever and inflammation. In inflammation there is similar derangement in the secreting and excreting functions: there is also sometimes similar derangement in the circulating function: but the derangement in the nervous and sensorial functions is seldom if ever similar: the derangement that does take place in these latter functions, while it is apparently different in kind, is certainly and invariably different in the order of its occurrence." 51.

Dr. Smith observes that we can now answer the question so often asked —"are fever and inflammation the same?"

"Fever and inflammation are not the same, because the term fever is appropriated to the designation of a certain number of events which occur in a certain series: the term inflammation, on the other hand, expresses another series of events, each event composing this train, succeeding each other in a different order: and the difference between the two series of events is precisely this difference in their individual phenomena and in their order of succession." 52.

The precise nature of the physical and physiological conditions of organs, both in fever and inflammation, are unknown. "We compare the events, and we see that they differ; and, since the use of names is to mark and express differences, it is right to distinguish these events by different terms." Out of the system of organs that are always affected in fever, some may be more and some less diseased—and from this diversity alone, the utmost variety may arise in the external characters of the malady.

"Thus, at one time, the spinal cord and the brain may be intensely affected: consequently the patient may be seized with violent pains in the limbs; with ferocious head-ache; with early delirium, which may rapidly increase to such a degree of violence as to require restraint: or, on the contrary, all the muscles of voluntary motion may be seized instantaneously with such a loss of energy that they may truly be said to be paralyzed: at the same time the sensorial faculties may be overwhelmed almost as completely as they are in apoplexy: thus may be formed one type of fever: and such a concourse of symptoms is actually found to exist: it ushers in the plague when it first stalks into a devoted city to sweep away its thousands and its tens of thousands.

"At another time the disease may seize with peculiar violence upon the organs of secretion, and especially upon those which belong to the digestive apparatus: hence the liver may suddenly pour forth an immense flow of bile, so vitiated in quality as to irritate and inflame whatever it touches, and so abun-

* Med. Chir. Journal and Review, Vol. I. p. 312. January, 1819.

dant in quantity as rapidly to diffuse itself over every part of the body, and to tinge almost every tissue and every fluid: at the same time the stomach and intestines may be involved in such acute disease that the powers of life may be exhausted in a few hours by incessant vomiting and unconquerable purging; thus may be formed another type of fever, and such a concourse of symptoms actually occurs in the yellow fever of the West Indies.

"Now we may witness a severe though a less violent affection of the spinal cord and the brain than occurs in plague. There may be present great pain in the back and limbs; intense head-ache; early and violent delirium; a burning skin; a quick and strong pulse; urgent thirst, and constipated bowels; or, on the contrary, there may be not pain of the head, but giddiness; not delirium, but stupor; not a burning hot, but a moderately warm or a cool skin; not a frequent and strong, but a frequent and feeble pulse. In either case we have a fair specimen of the common fever of our own country, the first forming the variety which may be termed acute, the second subacute cerebral.

"Now again we may witness a concurrence of symptoms very similar to the latter in the commencement of the attack, only that there is from the beginning greater prostration of strength; and a rapid increase in the derangement of the nervous and sensorial functions; together with a brown and dry tongue; a tender abdomen, and dark and offensive stools; thus may be formed another type of fever to which is commonly assigned the name of typhus." 55.

In each of these cases the most urgent symptoms are located in one set of organs, but the other organs of the febrile circle are as really, though not so intensely disordered. Severe affections of the brain will mask those of the stomach or vascular system, and vice versâ. How great then must necessarily be the diversity of symptoms presented by the different forms of fever—how incalculable the varieties resulting from difference of intensity alone!

"One degree of affection of the brain, for example, will occasion violent headache, constant watchfulness, great restlessness, a peculiar expression of the eye, and intolerance of light; in another there will be no headache, or none of which the patient will complain; there will be sleep though it be disturbed and unrefreshing; there will be no peculiar expression of the eye, and no intolerance of light. By one degree of affection the sensibility will be rendered preternaturally intense; by another it will be totally obliterated; one will produce violent delirium, another, only slight wandering, or unrefreshing slumber; one, violence requiring restraint; another, profound coma. In the circulating system the symptoms will alike vary. One degree will produce a quick, strong and hard pulse; another, a quick, small and feeble pulse; another, a slow and intermittent pulse. A similar diversity will be found in the temperature of the body; in one, the heat will be little changed; in another, it will be below the natural standard; in a third, it will be intense, and the organs of secretion and excretion will equally vary in the extent of their morbid changes." 58.

When to this variety are added the diversities occasioned by various stages of the diseased processes—by the previous state of the organs—by the reaction of one diseased organ on another, producing innumerable and ever varying combinations, can we wonder if the symptoms of fever appear to be countless! And as no two cases of fever can ever be precisely alike, it must be in vain, Dr. Smith thinks, to seek for the common phenomena of the disease in the external symptoms—hence, he observes, the proper object of pursuit in all these inquiries is the "real nature of the affection"—the symptoms being of consequence only as they are indications of the existence of that affection.

"It is then in the organs alone that we can find a perfect uniformity: but their condition is as fixed and invariable as the return of day and night. All the operations of nature are uniform. When, in any case, we have succeeded in discovering what the operation is, we see that it never varies. The same causes, under the same circumstances, always produce the same effects. The causes of fever, whatever they be, under the same circumstances, always produce the same conditions of the organs. In proportion as we ascertain with clearness and precision what these conditions are, we observe that they recur in all cases with the most undeviating regularity, and when our knowledge of them shall have become complete, it is probable that we shall find that they are as constant in their return as that of the sun after its setting, and that they no more change in their nature or progress than the sun deviates from its path." 60.

Much do we fear that the talented author is a little too premature in the assertion that "the condition of the most important organs in the various types of fever, as they occur in our own country, we do now know with precision." At all events, we apprehend that a great many years must elapse ere we shall be able to know, with precision, the condition of each organ *before death:*—that is, ere we can tell by the symptoms what are the changes going forward in the living body.

In the 3d chapter Dr. Smith exhibits a very able analysis of the symptoms of fever, more especially the synochus, or common fever of this country. We regret that we shall be able only to give a faint outline of the principal features of this interesting chapter. It is the more valuable, as many of the phenomena are drawn from the personal feelings of the author himself, while labouring under the disease. With a mind so trained to accurate observation and logical deduction, Dr. Smith's delineations are peculiarly valuable.

The first symptom which characterises the mild form of fever in this country (synochus mitior) is a loss of mental energy. This would always be noticed, if patients were capable of analyzing their sensations, or determining the order of their succession. Closely connected with this mental weakness, is the loss of energy in the muscles of voluntary motion, resulting in lassitude. Next in succession is that peculiar febrile malaise, which no words can describe, but which never can be forgotten by him who has once experienced it. It is much more distressing than pain. It is this which, in all febrile diseases, makes the patient wish for morning in the night, and long for night during the tedious hours of the day. To these symptoms positive pain soon succeeds—first in the back or loins, and then in the limbs. The countenance changes, and expresses dejection, the features being shrunk, and the tint pallid. The skin takes on increased sensibility to external agents, and diminished ability to resist their influence. Chilliness is felt even in a heated room. It arises from internal causes, and is not to be counteracted by external heat. It is not an actual deficiency of caloric in the body, for the thermometer applied to the skin or any other part, indicates the same heat as in health. The symptoms alluded to, are clearly referrible to derangement of function in the nervous system.

"There is as yet no affection of any other organ obviously or, at least, much developed. The circulating system, it is true, is just beginning to be affected. The pulse is no longer perfectly natural. It is more languid than in the state of health; sometimes it is also quicker: at other times it is slower; now and then it is scarcely changed in frequency, but its action is invariably weaker than in its sound state." 82.

We believe that accurate observation will shew the *coltemporaneous* derangement in the nervous, vascular, and secretory system, in almost every instance. We do not deny that the febrile cause is first applied to the brain and nervous system, and that the first morbid movements are there ; but we maintain that when derangement of sensation becomes appreciable, derangement of circulation and secretion will likewise be so. How indeed can it be otherwise with functions so intimately connected—nay dependent on each other?—But to return.

The respiration becomes affected—the breathing is shorter and quicker, especially if muscular movements be excited.

"The transition from the affection of the nervous and sensorial to that of the circulating and the respiratory systems is thus clear and striking. Physiology teaches us how closely these systems are connected, and how mutually they are dependent one upon the other, the closest observers and the ablest experimentalists candidly confessing that they are scarcely able to determine which is the least dependent, or the action of which the least necessary to the other's performance of its functions." 82.

Dr. Smith thinks that it may sometimes be many days before any other functions, except the sensorial, circulating, and respiratory, suffer in fever. This does not accord with our observation. All, or almost all the secretions are quickly if not simultaneously affected. The skin, according to Dr. Smith's own statement (page 81) is deranged in function in the primary movements of fever—and surely sensation is not the only, or even the principal function of the skin. Perspiration is equally important. Look at the kidneys, the intestinal glands, the salivary glands—they are all diminished in energy, with diminished power in the brain.

And now we see an opposite train of symptoms arise—namely reaction. The pulse becomes fuller, stronger, quicker than in health—the skin hotter.

"*Immediately the circulation is thus excited, the functions of secretion and excretion become deranged.* The mouth is now dry and parched; the tongue begins to be covered with fur; thirst comes on: the secretion of the liver, probably also of the pancreas, and certainly of the mucous membrane lining the whole alimentary canal, is vitiated, as is proved by the unnatural quantity, colour, and fetor of the evacuations; the urine likewise is altered in appearance, and the skin is not more remarkable for the sense of heat, than for that of dryness and harshness which it communicates to the touch. With the excitement of the pulse and the increase of the heat, the pain in the back and limbs and the general febrile uneasiness are much augmented.

"At this period, then, the fever is fully formed; the series of morbid phenomena is complete: any thing more that happens is referrible to degree and to duration, and must be the result of one or other of these circumstances, or of their combined operation." 85.

We certainly cannot coincide with Dr. Smith in the foregoing passage. No eloquence can persuade us, contrary to the evidence of our own senses, that secretion and excretion become deranged only when reaction commences in fever. According to our own observation those functions are deranged, though in a different manner, during the antecedent stage described by Dr. S. and all other writers on fever. It is true, the secretions and excretions are diminished—but they are also changed. Speaking of the incipient stage of fever previous to reaction, Dr. Parr, no mean authority, makes use of these words:—"The secretions are, in general, checked; the urine is watery; the mouth dry and clammy; ulcers no longer discharge, &c."

Neither do we think, with Dr. S. that when re-action takes place, "the series of morbid phenomena is complete." Excepting a few, and a very few cases of dreadful, quick, and fatal fevers, in the hotter regions of the earth, we have generally observed another stage, more or less defined, in which there was a kind of remission, marked by a state of sensorial energy, vascular action, and secretion different from the stages of chilliness and first accession of excitement. In intermittents, this is the most important stage of the three—and even in continued fevers, *at the commencement*, there is a morning remission and evening exacerbation, corresponding in kind, though very different in degree, with the train of phenomena described by Cullen and other systematic writers.

We need not dwell on the phenomena connected with the stage of excitement in fever, as increased pain in the head, increased sensibility to all external stimuli, augmentation of disturbance in the sensorial functions, depravation of the senses, watchfulness, &c. Our author avers that, in the fevers of this metropolis, the stage of excitement goes on uninterruptedly, or hardly ever with remissions, till the end, which is usually in fourteen days, when convalescence commences.

"One of the most remarkable circumstances connected with the ordinary fever of this country, in the present day, is the uninterrupted and perfect continuity of its phenomena. As long as the febrile state remains, nothing deserving the name of a remission is in general to be perceived. Occasionally, it is true, a slight increase in the symptoms may be observed towards evening, especially in the heat of the skin; but even this is not common, and it is scarcely ever great enough to deserve the distinction of being called an exacerbation. Much less is there any regularity in the accession and decline of such excitement. *In the great majority of cases not the slightest approach to an exacerbation and a remission can be distinguished from the commencement to the termination of the disease.* Yet the older writers speak of these events as if they were as palpable as the paroxysms of intermittent and as constant as the return of morning and evening. There cannot therefore be a doubt that the character of the ordinary fever of this metropolis is greatly changed from the character of that which prevailed two centuries ago; and the circumstances which have contributed to produce this change will be considered hereafter." 90.

We cannot pretend to put our experience in fever, as far as this metropolis is concerned, with the physician of the fever-hospital; but, as far as our observations extend, which are generally made upon a class of patients different from the inmates of a public institution, we have not observed those purely *continent* fevers alluded to by former writers, and now, by Dr. Smith's account, the prevailing ones of this capital. Even in the causus, the dreadful scourge of the West Indies, there is a remission between the storm of excitement, and the last melancholy stage of black vomit. As soon as the mornings get a little finer, and the evenings longer, we will invite Dr. Smith to a strict examination of a few patients at the Fever Hospital; and if we find them presenting the same phenomena at these two periods, we shall cheerfully admit that the worthy author has added a wrinkle to the horn of our knowledge.

Dr. Smith acknowledges that we are ignorant of the precise condition of the brain and other organs collectively affected in these mild forms of fever, because we have no opportunity of inspecting them, in consequence of the favourable termination, which is almost always the case.

"However this may be, and to leave for the present all matter of inference, and to keep strictly to the matter of fact, we do positively know that the mild forms of fever become severe in consequence of the supervention of inflammation in certain organs. Perfectly unknown as the nature of the primitive febrile affection at present is, yet that in the progress of the disease it does ultimately pass into inflammation is a fact, the evidence of which it is impossible to resist; although the same observation which teaches us this most important truth *teaches us also that the inflammatory action is always considerably modified by the febrile state.*" 94.

This is very rational pathology—and is almost exactly the same as has been advocated by all impartial observers of late years. Experience has taught us that fever is something different from inflammation—and that the great danger in fever is the supervention of inflammation—that death is almost always occasioned by the inflammation of some organ essential to life. Dr. Smith proceeds then to synochus gravior, which occurs under two degrees of intensity—the sub-acute and acute—and these two degrees he traces as they present symptoms of cerebral, thoracic, or abdominal affection. We are loth to pass the different sections unnoticed; and yet we must be very brief in our analytical sketches.

1. *Syn. Grav. with Subacute Cerebral Affection.* The accession, in these cases is the same as in the mild form already described, up to a certain period. But, at the time the head-ache diminishes in the former, it increases in the latter. Still it is often not severe. Practitioners are led into great error by apprehending no mischief in the head because head-ache is trifling, or altogether wanting. In the latter case giddiness is the substitute. In most cases, as long as the pain continues, the heat of surface remains much above par—especially about the head, and in particular parts of the head, corresponding with the seat of pain. The dull and heavy expression of the eye is greater than in synochus mitior—the conjunctiva becomes whiter and more glistening than natural, but sometimes more injected—the sensibility is preternatural when light is admitted, and this is not confined to the eye alone, but to the whole nervous system, especially the nerves of hearing. "Exposure to a glare of light and a loud noise would, alone, rapidly change a slight into the severest form of cerebral affection." The expression of the eye is indicative of suffering without the power to bear it. The face is sometimes flushed, often pallid. The want of sleep is distressing and the restlessness constant. Delirium now appears, though not invariably, and is seldom violent or long-continued. The pulse, the tongue, the evacuations, meantime, may not differ materially from those of the mild form of the fever.

Such is the train of symptoms for eight, ten, or even fifteen days, about which a remarkable change takes place, and an entirely new train of phenomena supervenes, presenting a striking contrast, according as the patient is destined for life or death. *If for life*, a balmy sleep takes place, from which the patient awakes a new being, and this change is easily recognized by the experienced eye, even of the nurse. The febrile symptoms are all mitigated—the amelioration increases—and convalescence is soon established. *If for death*, the head-ache often disappears, or at all events diminishes—the giddiness is no longer perceptible—the sensibility is obtunded—the mind becomes more dull and heavy—the patient may still be roused, but falls back into a state of stupor—delirium or incoherent muttering takes place—the pulse increases in frequency and is weaker—signs of disease in the chest and abdomen now present themselves, as purely cerebral fever from beginning to end is rarely to be seen. The tongue becomes more loaded and is often dry, though the thirst diminishes. From such a state recovery is rare, though it sometimes does occur. In general, things go on from bad to worse, and the patient dies exhausted. To the foregoing more common symptoms, there are sometimes others more uncommon added—as muscular tremor, sudden screaming, rolling of the head upon the pillow, jactitatio, floccitatio, involuntary discharges, &c.

In the acute cerebral affection of synochus gravior, the history is precisely the same, excepting that the symptoms are more severe, and their progress more rapid. One remarkable modification of the pulse in this dangerous grade of fever, is the slow and intermitting pulse. This, in conjunction with intense pain in the head or back, in the outset of fever, is a very ominous phenomenon, and indicates great severity of disease in the sequel. If active measures are not taken, the disease will be beyond control in a day or two, and the patient will die before the fever, in mild cases, would have time to form.

Cases in illustration of the acute and subacute affections in the two grades of fever are next detailed by the author, which we must pass over. Three sections follow on synochus gravior, with thoracic, abdominal, and mixed affections. In respect to the first, Dr. S. avers that there is probably "no case of fever, however slight, in which the mucous membrane of the bronchi remains in a perfectly sound state." This state of disease will be accurately described under the head of pathology. Whenever the thoracic symptoms are sufficiently intense to become prominent, and especially when they occur early in disease, they always greatly aggravate the general febrile symptoms. In this case two things happen—the proper febrile symptoms are modified—and new symptoms are added to the train. These new and peculiar symptoms are as follow.

"Pain in the chest, sometimes severe, sometimes only slight; sense of stricture or dyspnœa; inability to expand the chest by a full inspiration without pain or uneasiness; cough frequently aggravating the pain; sometimes dry, sometimes accompanied with frothy mucous expectoration. Respiration sometimes slow and heavy, at other times, on the contrary, short and quick; never natural: perhaps the physician may detect thoracic disease in the more obscure, and measure its extent in the more obvious cases, by observing the manner in which the patient breathes, better than by any other single means. The altered respiration is very frequently accompanied with that peculiar noise in breathing which is termed 'mucous rattle.'

"The pulse, in the commencement of this open and decided chest affection, may not be above 80 or 90; it is hardly ever sharp; it is generally weak; now and then it is full and of good strength; but whatever other character it may possess, it is almost always soft. In a few days, as the disease advances, it uniformly rises in frequency and becomes weaker. Towards the end of the disease it is almost always hurried and feeble, although cases occasionally occur in which it is observed at this period to become suddenly slow and intermittent. The tongue is usually foul; commonly moist; but, in severe affections and in their advanced stage, it sometimes becomes dry. The skin is often moderately warm, but it is never intensely hot: it is much more common for it to be cool, and to be of a more dusky colour than natural.

"Such are the usual conditions of the respiratory and circulating systems and of the tongue, the great index of the state of the mucous membrane of the alimentary canal, when the thoracic affection increases so as to become prominent and acute. The manner in which it influences the cerebral affection is commonly by hastening the period at which the pain of the head lapses into confusion and stupor. Early insensibility, assuming the form of a muddled or exceedingly confused state of mind, is a very constant symptom of more than ordinary thoracic affection. Accordingly, the delirium which succeeds or which accompanies this state is always low muttering talkativeness, or incoherent wandering, rather than violent delirium, which last is seldom, if ever, found in combination with severe thoracic disease. The pathological condition of the lung perfectly accounts for this modification of the condition of the brain, as will be shewn hereafter." 123.

The cases detailed in reference to this section we pass over.

*Synochus Grav. with Abdominal Affection.* In synochus mitior, our author thinks the mucous membrane of the stomach and bowels is but slightly affected; "but that it is really diseased, even in the mildest cases, we have sufficient evidence, in the invariable derangement which takes place in the functions of the organ throughout its whole course, from the mouth to the anus, and in the constant vitiation of its secretions and excretions."

"In the severer forms of fever on the other hand, in the great majority of cases, the affection of the abdomen becomes prominent, and whenever it does so it aggravates all the other febrile symptoms, and adds greatly to the danger of the disease." 128.

Abdominal affection, Dr. S. observes, may be severe from the commencement, and give early indications of its existence—or come on at a subsequent stage of the disease when the symptoms will be materially different. In the *former* case, in addition to the ordinary phenomena, there will be nausea, retching, or even vomiting—symptoms which, in decidedly cerebral cases, Dr. S. avers, are seldom present. When they are so, they indicate a severe abdominal affection, and it will be well if the practitioner is aware of this circumstance.

"At this early period the bowels are commonly constipated, and on inquiry *it will be found that they have been so for some days previously to the attack of fever*; but in a day or two after the commencement of this attack they fall into the opposite state and are looser than natural. The concurrence of nausea, retching, vomiting, and purging in the commencement of fever is a certain proof that severe abdominal affection is present, and if not actively treated and effectually checked at this early stage, it will soon render the case formidable, if not hopeless." 129.

The passage which we have marked in Italics, will bear us out in our observation that the secretory system is affected coeval with the other systems involved in fever. According to Dr. S. the abdominal secretions are locked up even before the attack of fever. This affection, when thus open and primary, is often attended with another symptom, namely, pain of the abdomen, attended, in exquisitely marked cases, by purging. But pain is not to be expected in all cases where there is serious disease going on in the abdomen. It is often absent in such cases:—but pressure of the epigastrium generally renders it unequivocal. Even when pain ushers in the abdominal affection, it usually diminishes after a certain time, and ceases altogether after the tenth day. Dr. Smith, and all accurate observers, have remarked how little patients will complain of pain in the abdomen, at a time when the slightest pressure on the epigastric region cannot be borne. This is a striking feature in the advanced stages of the disease. The following passage is certainly deserving of attention.

"There is thus a remarkable coincidence between the progress of the symptoms in the abdomen and in the head. We have seen that however intense the cerebral affection, the pain of the head which accompanies it diminishes after a certain time, and in a day or two after it has begun to diminish, ceases altogether. In like manner the pain which ushers in an acute abdominal affection diminishes after a certain time, and soon wholly disappears. After this period, therefore, we should have no more indications of abdominal than we have of cerebral pain were the intestines, like the brain, enclosed in a bony case. When an organ can be touched, it gives us an additional and an invaluable means of ascertaining its morbid condition; and this is one reason why that condition is commonly so much more certainly known in surgical than in medical diseases. What the result would be, could we press the brain as we can the abdomen, after its sensibility is so much diminished as to cease to occasion pain, we do not know; but it would be a bad use indeed to make of the additional means afforded us of ascertaining the condition of the intestines, were we to allow the additional information we thus gain, to obscure our perception of the perfect analogy there is in the progress of both affections. We know that, as the disease advances in both, the pain ceases; but, in the one case, we have the means of ascertaining that there still remains preternatural tenderness on pressure, as in ordinary inflammation, which we are without the means of discovering in the other: still the important practical fact afforded by the history of both is the same, that disease having reached a certain point, the pain diminishes; and having advanced still further entirely disappears." 132.

As the pain lessens or ceases, the purging advances. This phenomenon succeeding to constipation and pain, "affords an infallible indication of abdominal disease." The tongue affords another valuable diagnostic guide. It is preternaturally red—sometimes of a bright and vivid colour throughout—at others only at the sides or tip. The body is often loaded with fur, of a dirty white, yellow, or greyish colour. The papillæ appear enlarged, red, and elevated. As the intestinal disease advances, the vivid colour diminishes, and the tongue becomes dry, while the teeth become incrusted. The pulse is apparently free from any marked character in this complication of fever, and ranges usually from 80 to 100, beyond which it seldom goes, being generally soft. Sometimes, at an advanced period of the disease, the pain and purging both cease, and the motions appear healthy. If this be accompanied by a general amelioration of the other symptoms, it is a sign of recovery—if the other morbid phenomena continue, then it is a fatal symptom.

But there is another form of the disease which it is extremely difficult to discover, being attended with much less striking symptoms. "It steals, along its fatal course with a step as silent as it is sure; and the destruction that marks its track is oftentimes alike unfelt by its victim, and undiscovered by his most watchful guardian." It does not attack until the sensibility is greatly diminished, in consequence of cerebral disease. Pain on pressure therefore is not felt—and there is often no purging. The tongue is generally red at the edges and tip—loaded in the middle. The pulse usually runs up to 120. Recoveries do sometimes, though rarely, take place, even from this form—the favourable sign being *an increased tenderness of the abdomen on pressure.* This does not indicate increase of disease in the abdomen, but decrease of that in the brain.

The synochus gravior, with mixed affection, next occupies our author's attention, and the following extract will shew that it is not necessary to dwell on this part of the work.

"Since it has been repeatedly stated in the preceding pages that, in every case of fever, the brain, the lungs, and the abdomen are diseased, it may appear objectionable to call any particular class of cases mixed, because, according to the very nature of fever, all must be of this character. But for the same reason that we have designated one class of cases cerebral, another thoracic, and a third abdominal, namely, to mark prominence and intensity of affection, it is right to distinguish a fourth, in which all the three systems of organs are simultaneously affected with an equal, or nearly an equal degree of intensity. The term mixed is therefore by no means employed to intimate that the cases not comprehended under it are unmixed, but merely to point out a fact of great practical importance, that cases do occur which are neither in an exquisite degree cerebral, nor thoracic, nor abdominal, but which, at one and the same time, afford the most exquisite specimens of all the three.

"From this account of the sense in which the term is employed, it must be obvious that it will include the severest cases that can occur. If a patient be affected with intense cerebral disease he may be in great danger; but if he be affected with an equally intense thoracic disease his danger must be doubled; and if to this be added an equally intense abdominal disease it must be trebled. And accordingly these are just the cases which bid defiance to the most skilful and vigorous measures which the medical art can employ to control them; which seize upon their victim with a force which no human agency can resist nor counteract; which in malignant epidemics destroy life in a few hours or in a single hour, and in ordinary seasons in a few days." 143.

But whatever be the number of organs simultaneously affected, the nature of the affection in each is always the same. Disease in the brain is the same, whether the brain be alone prominently affected, or in common with the chest, the abdomen, or both. In like manner the symptoms, when any are present, are essentially the same, whether the disease exist alone, or whether it be complicated with several others. It would therefore be useless to detail the symptoms which occur in the mixed cases, since they must only be repetitions of those which have been already enumerated.

### Typhus.

The 4th chapter of the work is on Typhus, which, like Synochus, is divided into the *Mitior* and *Gravior*—two degrees of intensity merely of the same affection. Nay, the typhus itself is only synochus in an aggravated form.

"The appearance of a person labouring under typhus is so different from that of a person affected with synochus, that no one ignorant of the disease, who saw these two patients for the first time, would believe that both were afflicted with one and the same malady. And yet dissection after death demonstrates, that the physical condition of the organs is precisely the same in both; and careful examination of the symptoms during life, shews that they are really identical, both in their nature and their succession, however, at first view, they may appear to differ. *The difference between these two diseases arises entirely from a difference*

*in intensity;* still this difference produces a very important modification in the character of the disease; important, because it materially affects both the safety of the patient, and the nature of the remedies that are best adapted to rescue him from his danger." 149.

From this passage it will be evident that Dr. Smith, while he labours to simplify the *nature* of fever, is tolerably complex in the *forms* dependent on degrees of intensity. Thus the typhus, which is acknowledged to be merely a higher grade of synochus, is first divided into mitior and gravior, and then subdivided into cerebral, thoracic, abdominal, and mixed. We are told that "the symptoms which denote this affection (typhus mitior) are perfectly similar to those which have been stated to characterize it in synochus, but they undergo certain modifications, the true nature of which appears to have been greatly mistaken," and this mistake seems to Dr. S. of such magnitude, that he thinks whosoever shall effectually correct it "will do the greatest possible service to medicine." This being the case we shall introduce a rather long extract, in order that the author's views may not be garbled or misunderstood by abbreviation.

"1. There can be no question that, from the very first commencement of the attack, as well as through the whole course of the disease, the prostration of strength, both physical and mental, is greater in typhus than it is in synochus. This greater loss of energy is indicated by every sign that can be conceived to denote it. The loss of power in the muscles which support and move the body is oftentimes so complete, as to be most alarming to the patient and his friends, while the contrast between the vigor and the torpor of the mind, in the course only of a few hours, is most striking. From the full and active exercise of its faculties, it becomes, in that short space of time, quite incapable of performing any intellectual operation. It is confused and stupid, always in a greater degree than in synochus, and sometimes to such a degree, even on the very first day of the attack, as to excite the utmost apprehension in every one around the patient who takes any interest in his fate.

"2. The chilliness is, upon the whole, greater and longer-continued than in synochus: yet there is less constantly shivering, and the heat, when it succeeds this state of chilliness, is seldom as great as in the latter; while there are cases in which the heat never exceeds the natural standard.

"3. The febrile uneasiness is greater; the restlessness is incessant; the face is pallid; the features are shrunk; the expression of the countenance is most peculiar; it is strikingly indicative of weakness and suffering; the experienced eye can tell at a single glance even at this early period, to which of the two types that countenance belongs. The pulse is always weaker and more rapid than in the corresponding stage in synochus.

"4. There are cases in which the pain of the head is equally severe as in synochus: but this may be justly considered as rare. In general it is less acute. Dullness, confusion, stupor, giddiness, are more common than severe pain, and are often the substitutes for it. Though some degree of pain be generally present, yet it is by no means uncommon for one or more of these sensations to occupy its place completely. Question the patient as much as you please, and he will tell you that he has no pain; but it is evident, from his aspect and his manner, that he has little sensation of any kind. The eye is dull, heavy, stupid, without lustre: the old English word 'lac-lustre' expresses its character truly and strikingly. But it is remarkable, that while the pain in the head is only slight, the pains in the back, loins, and extremities, and, as the patient himself says, in the bones, are severe.

"5. When pain is present it diminishes sooner and disappears more completely than in synochus: when it is not present, the advancement of the disease is indicated by increasing insensibility, and by the rapid transition of dullness or confusion into a state of stupor approaching to coma. The eye is already muddy, and it soon becomes injected and suffused. The skin over the body is generally warm, sometimes hot: over the head it is often hot. The face is usually pallid, but the pallidness frequently alternates with flushing. The change of dullness into insensibility more or less profound sometimes takes place as early as the second or the third day: it is seldom that it is as late as the seventh or the eighth: it is postponed, when not prevented, by active and appropriate treatment.

"6. There is little or no sleep; the restlessness is great; there may be no violence; but there is abundance of inquietude.

"7. Delirium is more constantly present than in synochus; and when it comes it comes earlier: its presence is not unusual as early as the sixth or the seventh day; and it may appear still sooner, but that is rare. It consists of low muttering incoherence rather than of loud and violent talkativeness; and is expressed in moaning rather than in screaming.

"8. The connexion between delirium and muscular tremor, between muscular tremor and subsultus tendinum, and between both, and the passing of the stools and the urine unconsciously, has already been pointed out. Like delirium, muscular tremor is much more constantly present in typhus than in synochus; and its relation to delirium is so close that it is sometimes observed to supervene on the very same day; frequently on the day following; and, if it appear at all, it is seldom longer absent than the third. Its degree likewise is commonly in proportion to the violence of the delirium; and though early and great delirium may appear without it, yet it very rarely appears without delirium; and in general all these symptoms form one series or train; pain disappearing, confusion of mind increasing, muttering incoherence supervening, and muscular tremor and involuntary and unconscious stools rapidly succeeding.

"9. In the commencement of typhus the pulse is sometimes of good strength, and it may not exceed 90 in frequency; but as the disease advances it uniformly becomes weaker, smaller, and quicker; so that death rarely takes place before it has reached 120. In the severer cases it is weak, quick, and easily compressed at a very early period.

"10. The respiration is often not very obviously affected, but if it be attentively observed it will usually be found to be shorter and quicker than natural.

"11. The tongue is always foul on the first or second day; it seldom continues moist longer than three or four days; it is often quite dry as early as the fourth, especially on the body and at the root; the apex and the edges sometimes remain moist a day or two longer; but in a short time the whole tongue becomes perfectly dry and of a brown colour; as the disease advances the colour often changes to a darker and darker hue, until it becomes quite black; it is then frequently fissured into deep chaps, while the lips and teeth soon become covered with a black sordes. Were the sensibility not greatly altered, such a condition of the mouth and tongue must be attended with insatiable thirst; yet thirst is often not felt, although at other times it is considerable.

"12. In the early stage of typhus the skin is frequently hot; as the disease advances the heat lessens: through the greater portion of the disease it is moderately warm; towards its termination it becomes cool, and some days before death it falls below the natural standard. It is always of a darker colour than in synochus: the whole surface is of a dull and dusky tinge. Sometimes it is covered with dun-coloured petechiæ, at others with petechiæ of a florid colour.

"13. During its progress, erysipelas, first appearing on the face, then extending over the scalp, and often down the shoulders and back, is very apt to occur. Excoriation on the back and hips often form sloughing sores of great malignity and extent, while enlargement, inflammation and suppuration of glands situated in different parts of the body frequently appear.

"14. Typhus terminates much earlier, whether favourably or unfavourably, than synochus; if it terminate unfavourably death frequently takes place as early as the 10th or the 14th day, although if early and appropriate treatment be employed, the force of the disease is sometimes so much lessened that it is as protracted as synochus.

"Towards the termination of the disease more or fewer of the symptoms which it has been stated occasionally to occur in synochus, supervene; but, as these depend upon particular conditions of the brain, they will be detailed under the pathology." 155.

In respect to typhus gravior, we are happy to have Dr. Smith's authority for pronouncing it to be defunct in London—at least he has seen no example of it, either at the hospital or in private practice. All those examples which approached to the descriptions of the disease, as left us by Huxham, and even Cullen, consisted of the mixed cases of typhus, to which allusion has been made. All these examples have appeared referrible to two classes—one, in which the arterial action is excessive—the other in which it is oppressed, or rather overwhelmed. In the *first*, the patient lies insensible, with delirium ferox, rapid and panting respiration, tender abdomen, dry, black tongue, quick weak pulse, and pungent skin. In the *second*, he lies insensible also, with cold and dusky skin—swollen and livid countenance—heavy and oppressed breathing—pulse scarcely perceptible, and so rapid as to be innumerable. In this state the patient is almost as completely paralyzed as in the worst apoplexy, and the attack is nearly as rapidly fatal. Dr. S. observes that such forms of fever have been considered as exquisite specimens of diseases of debility; but to this he cannot subscribe. Where, says he, is the debility?

"Not in the disease, for that is of giant strength; not in the patient, for remove, if you can but remove, a part of the load that oppresses him, and instantly an intensity of action will be set up in the whole system, perhaps as great as it is capable of exerting, and certainly greater than it is capable of sustaining without the most imminent danger. The brain is overwhelmed by the intensity of its affection; the energy that should animate the system, and of which it is the great source, is withheld; but that energy is suspended, not destroyed; and the debility which seems to be the result is not real, but apparent, not direct, but indirect. The giant that lies prostrate on the earth, mastered by superior power, has still a giant's strength, though he do not at that moment put it forth: give him but the chance of throwing off the load that keeps him down, and he will soon shew you that he is not weak." 165.

It is remarkable, though not, we think, inexplicable, that the pathology of these intense cases should present a complete contrast to that of the less severe affections. "No morbid appearances are visible in the organs which are capable of accounting for death. There are signs of vascularity: the vessels are turgid with blood, and consequently the organs on which they are spent, are in a state of congestion." The reason why the products of inflammation are not found is, that there was not time for their formation.

We have one more chapter to analyze before we close this first article—and it is an important one, namely, the PATHOLOGY of fever.

"By carefully observing the symptoms in a large number of cases, we at length become acquainted with all the important symptoms that arise: by carefully examining the organs after death in a large number of cases, we gradually learn all the important changes in structure which they undergo; and by comparing, in all cases, the morbid symptoms with the altered states, we acquire in the end the power of ascertaining, with a high degree of probability, the presence of an event which we cannot see, by the presence of its sign which we can see." 177.

This would be true if the incipient changes were visible or tangible in their nature, and if they could be laid open in the early stages of fever. Our author confines his inquiries to the pathology of the solids, believing that the changes in these, are not only of far greater importance, but invariably antecedent to those in the fluids.

### I. External Appearances after Death.

The skin is more dusky than natural—sometimes studded with petechiæ, or even purple blotches. The body appears emaciated—the muscular fibre is very dark—and this darkness extends even to the viscera.

### II. Morbid Appearances in the Head.

The arachnoid membrane is more frequently affected than either of the others. It is either more vascular than natural, or thickened, opake, and milky—when in the latter state, there is generally a gelatinous fluid beneath it. There are often adhesions to the membrane above and below. The dura mater is less constantly changed in appearance, but it is sometimes more vascular than natural, or detached from the cranium in some places by effused fluid.

The pia mater is seldom changed in structure, but is generally preternaturally vascular. In like manner the theca vertebralis is often vascular, and contains more fluid than natural.

The brain itself is seldom perfectly sound. The changes consist of an altered state of its substance, cavities, or both. The most usual change in its substance is increased vascularity, sometimes on the surface, sometimes more deeply situated, existing in all degrees from a faint blush to a vivid redness. Mollescence and induration are occasionally seen after fever—sometimes partial, sometimes more general. Abscess in the substance of the brain does occur, but very rarely. The cerebellum is always found softer than the cerebrum. The morbid changes in the cavities are excess of secretion—varying from a drachm to several ounces. This excess of fluid is still more frequently found between the membranes than in the ventricles—especially between the arachnoid and pia mater. In this latter locality it is gelatinous—elsewhere lymphous. Excessive vascularity and increased secretion are never co-existent—the latter being the effect of the former, the cause ceases when the effect is fully produced. The substance of the spinal cord is seldom changed, either in vascularity or consistence. The morbid appearances are chiefly confined to the coverings, and are similar to those in the coverings of the brain.

### III. Morbid Appearances in the Thorax.

The mucous membrane of the bronchi is the most frequently affected, whatever be the type or degree of fever; and this affection, according to our author, is also "the most characteristic of the febrile state."

"Its disease is specific and uniform. It consists of preternatural redness. The character of this redness distinguishes it from that which is observed in ordinary inflammation. It is uniformly and strikingly darker, the difference in colour being precisely that which subsists between venous and arterial blood. This darkness of colour apparent in the bronchial lining, increases in degree as the tubes of the bronchi diminish in size ; while it may be only just discernible in the large trunks, the colour may be nearly black in the minute branches. This change in the natural colour of the membrane is indicative, not only of an increase in its vascularity, but of alteration in its structure. It is almost always attended with a preternatural thickening of its substance, as is demonstrated by cutting through the tube and reflecting the membrane. The tubes themselves contain more or less fluid, which consists of mucus, mixed with pus. Analogous to what has been stated with regard to the vascularity of the brain and to its secretions, when the quantity of secretion contained in the bronchial tubes is great, the degree of vascularity apparent in the membrane is lessened." 185.

In scarlatina the morbid changes are somewhat different. The mucous membrane covering the trachea, larynx, amygdalæ, and soft palate, is inflamed—the redness is of a more vivid colour than in fever without eruption —being similar to the characteristic colour of the scarlatina tongue.

When in every other respect healthy, the substance of the lungs in fever is so constantly found either gorged with blood, or infiltrated with serum, that these changes would seem to form essential parts of the morbid phenomena.

The pleuræ exhibit various degrees of vascularity, from the faintest blush to the most intense phlogosis—and every extent of adhesion, from the smallest point, to the complete obliteration of the thoracic cavities. The usual products of inflammation are likewise found equally varying in degree. The parenchyma of the lungs, besides engorgement and infiltration, presents hepatization and tuberculation in every variety and degree—ulceration and abscess—hæmorrhagic and calcareous depositions—and enlargement and melanosis of the bronchial glands. These changes, however, are not peculiar or essential to fever.

### IV. MORBID APPEARANCES IN THE ABDOMEN.

The viscera always appear darker than natural, and generally more vascular. Several of the organs are affected in a uniform and peculiar manner —but the mucous membrane of the small intestines, especially of the ileum and cæcum, suffers most. The lesions are vascularity, thickening, ulceration. The vascularity or inflammation is often confined to the inferior extremity of the small intestines, when not the slightest deviation from healthy structure can be perceived in any other part of the canal. The second stage of disease consists in thickening of the membrane, or in deposition of matter beneath it in the situation of the mucous glands. The glands themselves are found in all states and stages of disease, from the least to the greatest enlargement, and from mere abrasion of surface to entire ulceration of surface.

The third stage is ulceration, which may supervene on either of the other affections, but the ulcer will not be the same in both cases.

"If ulceration take place while the mucous coat is in a state of simple vascularity, the ulcer will in general be extensive but superficial; its surface will present a smooth appearance, and its margin will be regular and defined : if, on the contrary, it occur after thickening of the membrane or enlargement of its glands, its characters will be just the reverse : it will be less extensive, but more deep, because it must penetrate a mass of adventitious matter before it can reach the other coats; and, for the same reason, its margin will be more elevated and its surface more ragged. It is in this form of ulcer that perforation of the intestine generally occurs; in which case the mucous and muscular coats alone are ulcerated: the peritoneal gives way from gangrene.

"Whenever the mucous membrane is ulcerated, whatever be the form of the ulcer, the corresponding portion of the peritoneal coat is more vascular than natural; and perforation must be attended with inevitable death, on account of the extensive and intense peritonitis excited by the escape of fæces into the peritoneal cavity." 189.

The mesenteric glands are usually diseased in proportion to the other lesions already described. The pancreas, Dr. S. avers, is very constantly diseased in fever. It is always more firm than natural—the reverse of what takes place in the spleen. Sometimes it is nearly cartilaginous. As to the mucous membrane of the stomach, Dr. S. informs us that the uniform result of post-mortem examinations at the hospital is, "that the mucous membrane of this organ is less frequently, less severely, and less extensively diseased than any other portion of the same membrane." In no instance was ulceration found in the stomach. The liver is less frequently changed in structure than any other organ. The blood contained in it is always dark and fluid—its parenchyma is sometimes softer than natural—gall-bladder full of unhealthy bile, being either too pale and thin, or too dark and thick.

The preceding comprehend all the morbid conditions of the abdominal viscera which are peculiar to fever. The author then details a great number of cases and dissections illustrating the cerebral, thoracic, and abdominal pathology of the disease, and then addresses the reader thus:—

"And what is this pathology? What are the events, the detail of which has occupied us so long? The account of the pathology of fever is the history of inflammation, and the description of the individual changes that take place in the organs that constitute the febrile circle, is an enumeration of various products of inflammation which are formed within them. There is scarcely a fatal case of fever which does not afford, in one or other of the organs of that circle, some inflammatory product; there is no considerable number of fatal cases which does not furnish a specimen of every inflammatory product. And what are the severest cases of fever, and why are they the severest? With the single exception immediately to be stated, the severest cases are those in which, together with a severe primary affection of the nervous system, this inflammatory action is in the greatest degree of intensity, and is seated in the greatest number of organs; and they are the most severe, not only on account of the severity of the primary affection of the nervous system, but also because it is in them that the inflammation is the most intense, and because that inflammation attacks the system at one and the same time in the greatest number of points. From among the preceding cases, fix upon any one in which the powers of life were, from the commencement, the most completely overwhelmed, and in which they were the most rapidly exhausted, and when the brief struggle for existence is over, examine the changes that have taken place in the internal organs—what is it that is found? traces of inflammation, legible, deep, extensive; while, in almost every case, these traces are thus legible, deep, and extensive, in proportion to the apparent intensity of the fever, and to the rapidity with which it extinguished life." 324.

Unquestionably if pathology means morbid anatomy, the lesions must all be referred to inflammation and its products; but if pathology mean the whole morbid process of fever from beginning to end, the doctrine of inflammation will not hold good—even according to Dr. Smith's own shewing. In the succeeding section Dr. S. truly states that the functions of the brain and nervous system are the first, invariably, to be disturbed in fever; but of the nature of this primary functional disorder, we are ignorant. The phenomena of fever and inflammation, he thinks, prove "that the two diseases are not identical." The fever affection he regards as specific.

"This peculiar and specific affection appears to be much more analogous to the condition into which the nervous system is brought by the application of certain poisons, than to that which is proper to pure inflammation. The more closely and extensively the subject is investigated, the more clear and satisfactory the evidence becomes, that the great primary cause of fever is a poison, the operation of which, like that of some other poisons, the nature of which is better understood, and the action of which has been more completely examined, is ascertained to be upon the nervous system. How these poisons act upon the nervous system we do not know, nor can we possibly know, as long as we remain so profoundly ignorant of the nature of the action of the nervous system in the state of health.

"It may be considered then as established, that the primary morbid condition of the body, in fever, consists of an affection of the nervous system, which there is reason to believe is of a peculiar and specific nature, although that nature be at present wholly unknown." 337.

This peculiar specific, and unknown affection of the nervous system, having continued for some time, brings the vascular system into a morbid state—and this state passes, "sooner or later into *true* inflammation." The second event then, that takes place in fever, is inflammation. But this inflammation, Dr. S. thinks, "is never pure or simple." "The condition of the inflamed organs is never the same as that into which they are brought by mere phlegmasia:—there is always inflammation, *and something else*." "That *something else* is the unknown, but the peculiar and specific affection of the nervous system, which is invariably antecedent of whatever subsequent affection may take place." "This affection of the nervous system is not only the invariable antecedent of every other condition, but it is omnipresent with every other condition, and its presence is a most powerfully influential presence." This combination of fever and inflammation, modifies the latter very much indeed, as well as the treatment which is to be used in its cure.

It has been stated by our author that there are two exceptions to the universality of the presence of inflammation in fever—in the mildest cases, and in those that are rapidly fatal. In the first case, the vascular action does not appear to rise into that grade which offers the common phenomena or products of inflammation—and in the second case, there is not time for inflammation to manifest itself by the usual proofs. In all the intermediate shades and grades, he thinks there is inflammation as surely as in pleuritis. This guarded theory, he conceives, is not only the safe one in practice, but the only one that can explain the ratio symptomatum, and guide the methodus medendi.

"He who believes fever to consist of an affection of the nervous system alone, every other affection that may be combined with it being accidental, will rarely think of using the lancet: he who believes fever to consist of inflammation alone, and overlooks the presence of the nervous affection, will be apt to carry the employment of the lancet too far: he alone who embraces the view of both, brings within his own all the phenomena: he alone adopts a sound theory of the disease, and we now see that he alone is likely to be led to a sound practice." 342.

Here we must allow our readers as well as ourselves to draw breath before, in another, but short article, we complete the analysis of Dr. Smith's and Dr. Tweedie's works. This completion will be in the present quarterly number, and will not therefore break the continuity of the chain of investigation.

---

